# Identification of Inhibitors of the Disease-Associated Protein Phosphatase Scp1 Using Antibody Mimetic Molecules

**DOI:** 10.3390/ijms25073737

**Published:** 2024-03-27

**Authors:** Tamaki Kobayashi, Kazuki Yamazaki, Junki Shinada, Masataka Mizunuma, Kazuhiro Furukawa, Yoshiro Chuman

**Affiliations:** Department of Chemistry, Faculty of Science, Niigata University, Niigata 950-2181, Japan; f23a032a@mail.cc.niigata-u.ac.jp (T.K.); kazuki.yamazaki1204@gmail.com (K.Y.); soeg1120@gmail.com (J.S.); f21j007a@mail.cc.niigata-u.ac.jp (M.M.); furukawa@chem.sc.niigata-u.ac.jp (K.F.)

**Keywords:** protein phosphatase, adnectin, phage display library, Scp1, anticancer drug

## Abstract

Protein phosphorylation is a prevalent translational modification, and its dysregulation has been implicated in various diseases, including cancer. Despite its significance, there is a lack of specific inhibitors of the FCP/SCP-type Ser/Thr protein phosphatase Scp1, characterized by high specificity and affinity. In this study, we focused on adnectin, an antibody-mimetic protein, aiming to identify Scp1-specific binding molecules with a broad binding surface that target the substrate-recognition site of Scp1. Biopanning of Scp1 was performed using an adnectin-presenting phage library with a randomized FG loop. We succeeded in identifying FG-1Adn, which showed high affinity and specificity for Scp1. Ala scanning analysis of the Scp1-binding sequence in relation to the FG-1 peptide revealed that hydrophobic residues, including aromatic amino acids, play important roles in Scp1 recognition. Furthermore, FG-1Adn was found to co-localize with Scp1 in cells, especially on the plasma membrane. In addition, Western blotting analysis showed that FG-1Adn increased the phosphorylation level of the target protein of Scp1 in cells, indicating that FG-1Adn can inhibit the function of Scp1. These results suggest that FG-1Adn can be used as a specific inhibitor of Scp1.

## 1. Introduction

Protein phosphorylation is a reversible process tightly controlled by protein kinases and phosphatases. The phosphorylation of Ser or Thr residues in proteins accounts for 98% of total phosphorylation, and this process is reversed by Ser/Thr protein phosphatases through dephosphorylation [1]. Ser/Thr phosphatases are broadly classified into the following three families: phosphorylated protein phosphatases (PPP), metal-dependent protein phosphatases (PPM), and aspartate-based catalytic phosphatases (FCP/SCP) [2]. Scp1 is a member of the FCP/SCP family and contains a DxDxT/V motif and a hydrophobic pocket at the substrate-binding site [3]. The phosphorylated Ser5 (CTDpS5) of the C-terminal domain (CTD) tandem heptad repeat Y_1_S_2_P_3_T_4_S_5_P_6_S_7_ in RNA polymerase II (RNAPII) is known as the Scp1-targeting site, and its phosphorylation status is involved in its transcriptional activity [4]. Although CTDpS5 is known as the representative substrate of Scp1, it has recently been shown that Scp1 plays an important role in various biological activities, such as cell cycle regulation, signal transduction, and neuronal gene silencing, through the dephosphorylation of various substrates [5,6,7,8,9]. RAC-alpha Ser/Thr-protein kinase (Akt) has been reported to be a Scp1 substrate and is involved in signaling that activates angiogenesis [10]. In addition, the RE1-silencing transcription factor (REST) complex, also known as Scp1 substrate, which acts as a molecular switch for neuronal differentiation, is involved in neuronal gene silencing [8,9,11]. The dysregulation of REST has also been implicated in several neurological diseases, including Alzheimer’s disease, suggesting that Scp1 dysfunction is associated with these diseases [12,13,14,15]. Furthermore, Snail, a known inducer of epithelial–mesenchymal transition that promotes cancer metastasis and invasion, has also been reported to be a substrate of Scp1 [16,17]. These findings suggest that inhibitors targeting Scp1 may serve as lead compounds for nerve regeneration and as anticancer agents by inhibiting the dephosphorylation activity of Scp1 [18,19,20,21,22].

The development of inhibitors targeting Scp1 has been vigorously pursued, and several laboratories have reported the use of Scp1 inhibitors [23,24]. We also successfully developed peptide inhibitors targeting Scp1 using the phosphorylation mimic phage display (PMPD) method derived from the substrate recognition mechanism [25,26,27]. Peptide inhibitors are required to improve structural stability by introducing non-natural amino acids or structural restrictions for in vivo use, although they have been confirmed to specifically inhibit Scp1. On the other hand, rabeprazole is the only commercially available specific inhibitor of Scp1 [23]. However, its low inhibitory activity, on the order of μM against Scp1, as well as its low specificity for Scp1, since it also inhibits H^+^⁄K^+^ ATPase, raises concerns about the risk of side effects due to different target binding [28]. Several Scp isoforms (Scps) have been reported, including Scp1–Scp3, with similar structures and sequences in the substrate recognition pocket [23]. This suggests that it is difficult to develop small-molecule drugs, including rabeprazole, as isoform-specific inhibitors because of their narrow binding area to the common binding pocket of the isoforms. Therefore, the development of strong inhibitors with a broad binding surface for Scp1, as well as high affinity and specificity, is highly desired.

**Figure 1:**
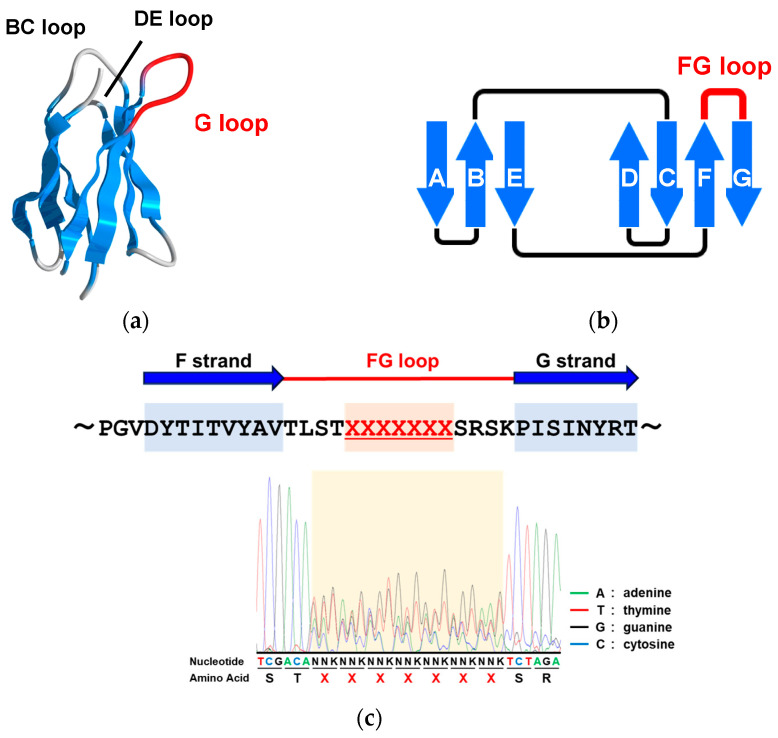
Construction of an adnectin-derived phage display library: (**a**) the structure of adnectin. The FG loop is known to be the longest loop in adnectin and plays an important role in target binding; (**b**) adnectin forms a β-sandwich structure consisting of seven β-strands. The structure of adnectin is similar to that of the target recognition site of antibodies; (**c**) sequence analysis of the FG loop of the constructed adnectin library.

Recently, antibody drugs have been widely used as therapeutic and detection agents for various diseases owing to their high affinity and specificity for their targets [29]. However, the disadvantages of antibody drugs with high molecular weights and high production costs have facilitated the development of artificial binding proteins, such as adnectin, nanobody, DARPin, and Anticalin, which have been investigated as alternative target-binding proteins [30,31,32,33,34]. Adnectin, also known as a monobody, is a target-binding macromolecule derived from the 10th type III domain of human fibronectin [35,36]. Adnectin is a small protein of approximately 10 kDa, with a molecular size of approximately 1/15 that of an antibody. The scaffold of adnectin, which resembles the target recognition site of antibodies, consists of a stable β-sandwich structure comprising seven β-strands, and it shows high thermal stability [37]. There are three target recognition loop regions, called the BC, DE, and FG loops, which can adopt various binding modes (Figure 1a,b) [38]. In particular, the FG loop, which is the longest and most flexible of the three loops, has been reported to contribute the most to target recognition and is used to fit into pocket structures or sandwich the target between two other loops [39,40]. These loop sequences have been incorporated into libraries, and many adnectin-derived binding molecules targeting proteins such as human αvβ3 integrin and Src SH3 have been reported thus far [41,42]. Our laboratory has also identified an adnectin-based molecule that binds to the breast cancer-associated protein PPM1D, a metal-dependent protein phosphatase [43]. Unlike antibodies, adnectin does not contain disulfide bonds, suggesting that it allows for expression and function in the cytoplasm, which is a reductive environment [44,45,46]. Taken together, the antibody-mimetic molecule adnectin can be used to recognize intracellular targets, unlike antibodies, and may be useful for the development of lead compounds as diagnostic and therapeutic agents, not only in vitro but also in vivo.

## 2. Results

### 2.1. Construction of an Adnectin-Derived Phage Display Library with a Randomized FG Loop

We generated adnectins using a randomized codon design {(NNK)_7_; N = A, T, G, or C; K = T or G} for each of the seven amino acids in the FG loop and fused them to the N-terminus of the M13 minor coat protein gIIIp. The constructed phagemid vector was introduced into *Escherichia coli* TG1-competent cells via electroporation to prepare the library. The sequence analysis of the *E. coli* library revealed that randomized sequences were introduced into the FG loop sequence (Figure 1c). Phage rescue was performed using the M13KO7 helper phages to construct an adnectin-derived phage display library. This phage library was screened for Scp1-specific adnectins.

### 2.2. Screening of Scp1-Specific Adnectin from the Constructed Phage Display Library

**Figure 2:**
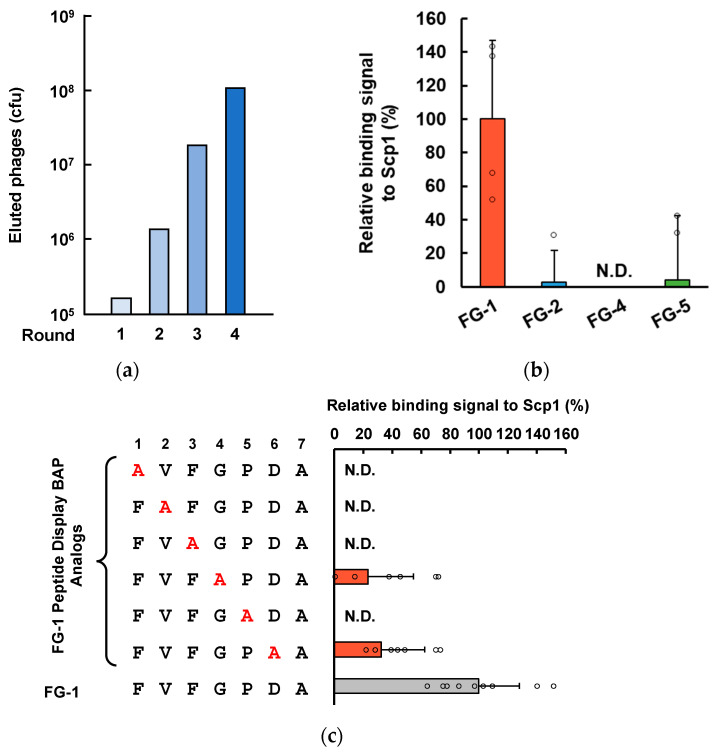
Isolation of Scp1-specific adnectin using a randomized adnectin-derived phage display library: (**a**) the process of screening for Scp1-binding phage; (**b**) binding analysis with FG loop sequence peptide-conjugated BAP (*n* = 4); (**c**) identification of the contributed amino acid residues to the binding of Scp1 by substitution Ala residue using peptide-BAP conjugates (*n* = 9). Amino acids subjected to replace Ala residue are colored in red. Analogs that showed binding signal to Scp1 are highlighted in orange. N.D., non-detectable.

To identify Scp1-binding adnectin from the adnectin-derived phage library, four rounds of biopanning were performed using Scp1-coated ELISA plates. The Scp1-specific adnectin-presenting phage was eluted by adding an excess of Scp1. The number of recovered phages increased in every round, suggesting the convergence of Scp1-binding clones (Figure 2a). 

Twenty-one adnectin-derived phage clones from rounds three and four were isolated and subjected to sequencing analysis. Sequence analysis of the 13 clones showed that the adnectin-derived phage clone with the FG-1 sequence was detected with the highest frequency, and FG-2 was also detected five times; however, FG-3 to FG-5 were detected only once (Table 1). These results indicate that FG-1 is the most promising candidate for the Scp1-binding sequence. Interestingly, sequence comparison of FG loops from isolated adnectin-derived phage clones revealed that Pro residues, followed by acidic amino acids on the C-terminal side, were highly conserved among the isolated phage clones, suggesting that Pro and acidic amino acids contribute to Scp1 binding.

Bacterial alkaline phosphatase (BAP) chimeras with a peptide sequence of the FG loop were generated to evaluate the binding ability of FG-1 to FG-5 peptides for Scp1. Expression analysis of BAP with FG peptides using an *E. coli* expression system showed that FG-1, 2, 4, and 5 displayed BAP expression, but FG-3 did not. This suggests that the FG-3 sequence may have an inhibitory effect on BAP expression in *E. coli*; therefore, we performed binding analysis without FG-3. Binding analysis using peptide-conjugated BAP showed that FG-1 peptide bound to Scp1 with the highest affinity, although FG-2 and FG-5 also showed weak binding, suggesting that these peptide sequences could recognize Scp1 (Figure 2b). 

To determine the contribution of a specific residue to the binding of Scp1, we performed Ala scanning of the FG-1 sequence. Ala scanning analysis showed that all Ala mutants decreased the binding ability, especially the Ala mutants of Phe_1_, Val_2_, Phe_3_, and Pro_5_, which completely cleared binding to Scp1 (Figure 2c). These results strongly support the results that FG-1 or FG-5 BAP within the (V/L)FxP motif, followed by acidic residues, binds to Scp1 and that hydrophobic and aromatic rings are important for binding to Scp1.

### 2.3. In Vitro Functional Analysis of Recombinant Scp1-Specific Adnectin

**Figure 3:**
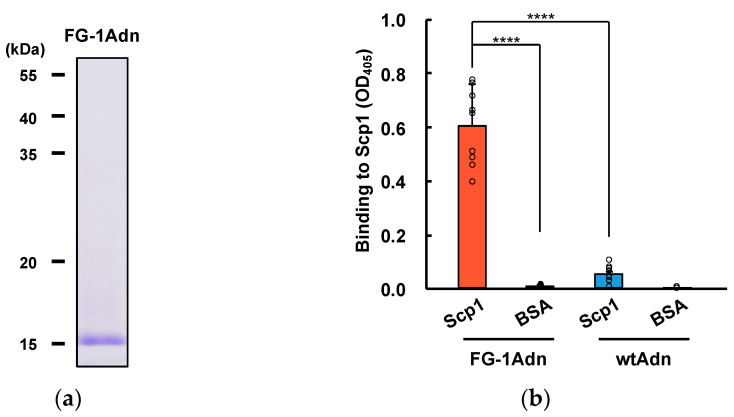

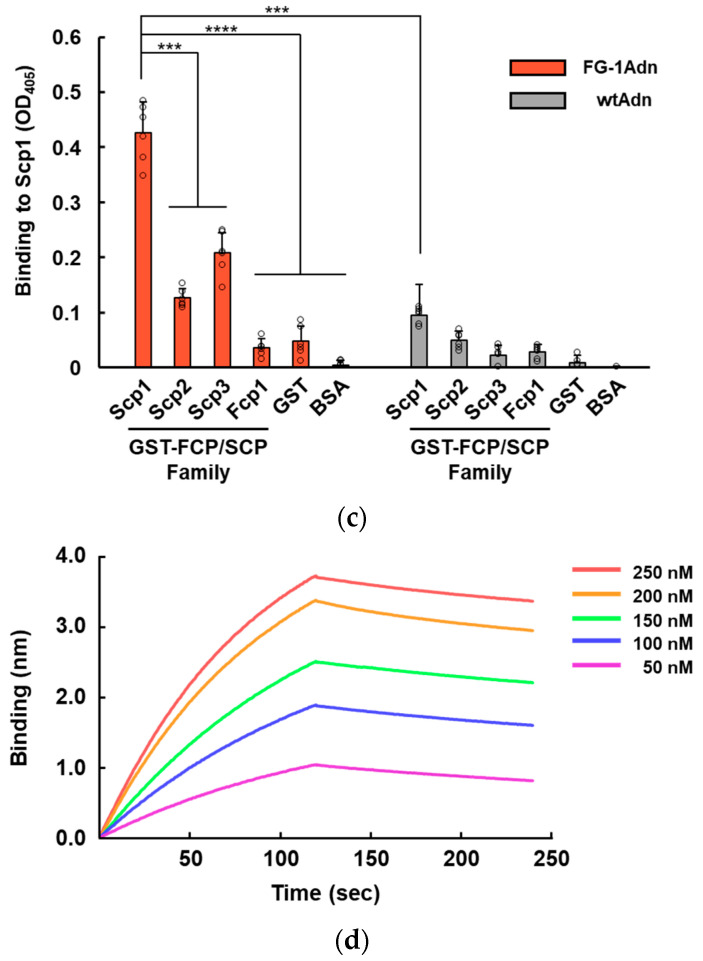
In vitro functional analysis of FG-1Adn: (**a**) SDS-PAGE of recombinant FG-1Adn targeting Scp1. The recombinant proteins expressed by *E. coli* system were recovered with high purity.; (**b**) the binding ability of recombinant FG-1Adn and wtAdn to Scp1 (*n* = 9). Statistical significance was asserted between the indicated adnectin and target proteins by two-way ANOVA with Tukey’s post hoc test. ****: *p* < 0.0001. The data are presented as the mean ± SEM; (**c**) specificity of FG-1Adn and wtAdn recombinant proteins in FCP/SCP family. Scp2 and Scp3 are isoforms of Scp1 (*n* = 6). Statistical significance was asserted between the indicated adnectin and target proteins via two-way ANOVA with Tukey’s post hoc test. ***: *p* < 0.001, ****: *p* < 0.0001. The data are presented as the mean ± SEM; (**d**) binding assays of FG-1Adn to Scp1 by biolayer interferometry. Scp1 was measured in the presence or absence of biotinylated FG-1Adn. The measurement was performed in the Advanced Kinetics mode of the BLItz system using streptavidin biosensor chips.

Next, we evaluated the ability of FG-1Adn, wherein the FG-1 peptide sequence was introduced into the FG loop of adnectin. FG-1Adn was expressed using an *E. coli* expression system, and the proteins were purified through the use of affinity chromatography. Recombinant FG-1Adn was analyzed using SDS-PAGE and confirmed to be highly purified (Figure 3a). Purified FG-1Adn was subjected to ELISA to analyze its binding to Scp1. This binding analysis showed that FG-1Adn bound significantly to Scp1 in comparison with wt adnectin (wtAdn), suggesting that the FG-1 peptide sequence binds to Scp1 (Figure 3b).

Then, we performed target specificity analysis using other phosphatases belonging to the FCP/SCP-type Ser/Thr phosphatase family. In this analysis, FG-1Adn showed the strongest binding to Scp1; however, it showed weak binding to Scp2 and Scp3, and no binding to Fcp1 was detected among the FCP/SCP family (Figure 3c). The Scps showed high homology, especially in the catalytic domain, where 81.1–84.9% of the residues in the FCP homology domain were conserved. However, Fcp1 showed low homology with all the Scp isoforms, maintaining a conserved sequence of around 19.9–22.8%. These results suggest that FG-1Adn binds to the highly conserved catalytic domain of Scps. In addition, the difference in binding affinity among Scps may be due to the contribution of the N-terminal region, which exhibits a unique sequence between Scps.

To further analyze the ability of the identified adnectin to bind Scp1, the dissociation constant of FG-1 was calculated using the BLItz system. Biotin-labeled FG-1 was fixed on a streptavidin chip, and the dissociation constant for Scp1 was calculated as *K*_D_ = 48.7 ± 0.09 nM (Figure 3d). These data strongly indicate that FG-1Adn binds to Scp1 with high affinity.

**Figure 4:**
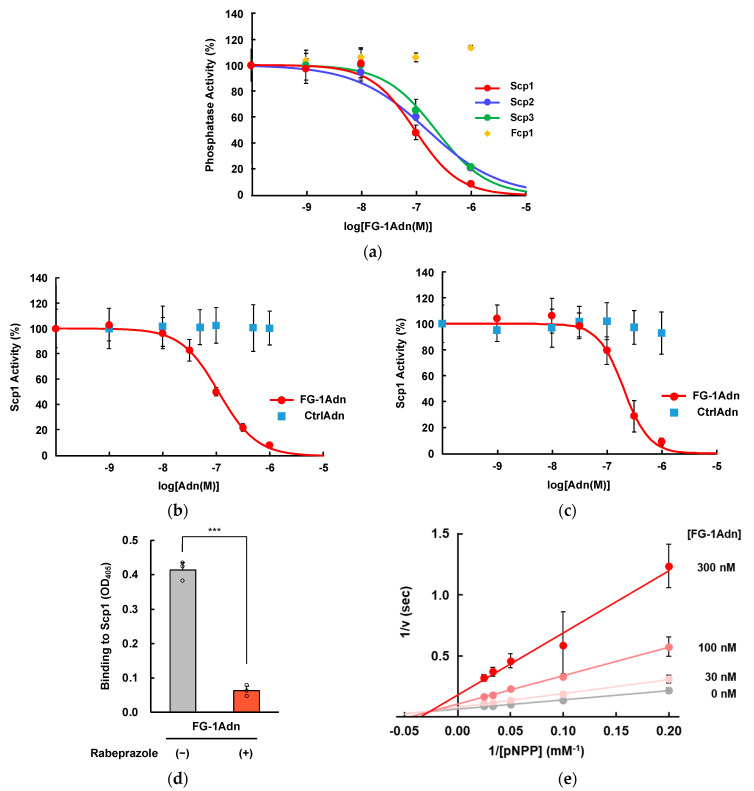
Inhibitory analysis of FG-1Adn against Scp1: (**a**) inhibitory effects of FG-1Adn inhibition against FCP/SCP phosphatases using *p*NPP as a substrate (*n* = 3); (**b**) concentration dependency of FG-1Adn inhibition against Scp1 using *p*NPP as a substrate (*n* = 9); (**c**) concentration dependence of FG-1Adn inhibition against Scp1 analyzed via malachite green assay using CTDpS5 peptide as a substrate (*n* = 9); (**d**) competition binding analysis between FG-1Adn and rabeprazole (*n* = 3). ***: *p* < 0.001; (**e**) Lineweaver–Burk plot analysis of FG-1Adn to clarify the inhibitory mode against Scp1 (*n* = 5).

Next, the enzyme specificity of FG-1Adn was analyzed in relation to the inhibitory effects against FCP/SCP type Ser/Thr protein phosphatases. The analysis of the inhibition concentration dependence of FG-1Adn using *p*NPP, which is widely used for phosphatase assays as a substrate, showed that the IC_50_ values for Scps were 96 ± 28, 161 ± 48, and 230 ± 84 nM, respectively, while no inhibitory activity was observed against Fcp1. Therefore, FG-1Adn showed the highest inhibitory activity against Scp1 among the FCP/SCP phosphatases as in the binding test (Figure 4a). These results suggested that the inhibitory effect of FG-1Adn occurs via direct binding by Scp1.

Then, to confirm the importance of the FG sequence of FG-1Adn in the inhibitory activity, inhibitory activity was compared with CtrlAdn using *p*NPP, and the CTDpS5 peptide, the phosphorylated Ser5 of the CTD in RNAPII, which is an endogenous substrate of Scp1. In the *p*NPP assay, FG-1Adn inhibited the dephosphorylation activity of Scp1 in a concentration-dependent manner and exhibited an inhibition ability with 112 ± 17 nM of IC_50, *p*NPP_ while CtrlAdn showed no inhibitory activity against Scp1 (Figure 4b). Inhibitory analysis of FG-1Adn to Scp1 using CTDpS5 as a substrate also showed a strong inhibitory effect with 152 ± 26 nM of IC_50, CTDpS5_, but not CtrlAdn (Figure 4c). These results suggest that FG-1Adn inhibited Scp1 through its own FG loop.

In addition, to explore the binding site of FG-1Adn to Scp1, a binding competition analysis was conducted with rabeprazole, which is known to bind to the hydrophobic region in the vicinity of the active center of Scp1. The results showed that rabeprazole almost completely inhibited the binding of FG-1Adn to Scp1 (Figure 4d). These data strongly suggest that FG-1Adn binds to the hydrophobic pocket of Scp1, similar to rabeprazole.

To further investigate the inhibition mode of FG-1Adn on Scp1, Lineweaver–Burk plot analysis was performed using *p*NPP as a substrate. The straight lines obtained from the inverse plot of each of the FG-1Adn concentrations almost intersect each other on the x-axis. (Figure 4e). These results suggest that FG-1Adn inhibits Scp1 in a manner similar to non-competitive inhibition. The binding of FG-1Adn to Scp1 is inhibited by rabeprazole, which binds to the hydrophobic region near the active center of Scp1, and by the non-competitive inhibitory binding of FG-1Adn to Scp1, strongly suggesting that FG-1Adn binds to the hydrophobic region near the active center of Scp1. 

### 2.4. Biological Functions of FG-1Adn in Cells

**Figure 5:**
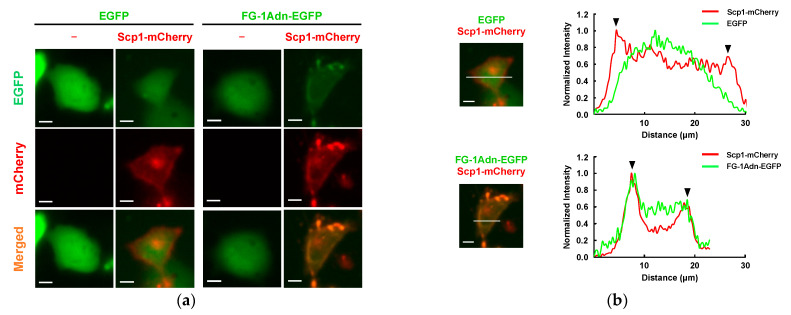

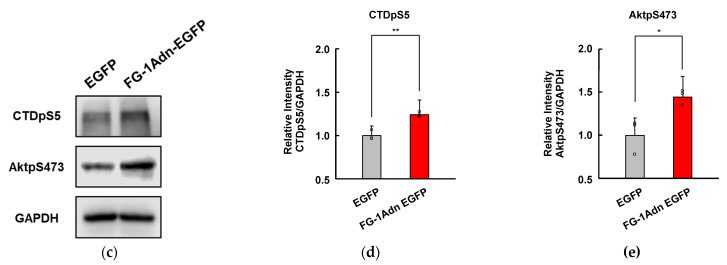
Subcellular localization and functional analysis of FG-1Adn in H1299 cells: (**a**) subcellular localization of FG-1Adn-EGFP (green) and Scp1-mCherry (red) was analyzed in H1299 cells. Scale bars, 10 µm; (**b**) quantitative analysis of subcellular localization of FG-1Adn-EGFP (green) and Scp1-mCherry (red). Fluorescence intensity was normalized using Image J. The black arrowheads indicate the plasma membrane on the cell surface. Scale bars, 10 µm; (**c**) Western blotting analysis of H1299 cells expressing FG-1Adn-EGFP was performed, and the inhibitory effect of FG-1Adn-EGFP on Scp1 was evaluated via dephosphorylation levels of CTDpS5 and AktpS473. GAPDH was detected using an anti-GAPDH antibody as an internal control protein. These experiments were performed as three independent experiments to confirm reproducibility; (**d**,**e**) quantitative evaluation of Western blotting analysis on CTDpS5 and AktpS473 from three independent experiments (*n* = 3); *: *p* < 0.05, **: *p* < 0.01.

Next, we assessed the intracellular regulation of Scp1 by FG-1Adn. The subcellular localization of EGFP-fused FG-1Adn and mCherry-fused Scp1 was analyzed in lung cancer-derived H1299 cells. A strong Scp1-mCherry signal was observed on the plasma membrane, as well as throughout the cell (Figure 5a). This result is consistent with previous reports showing that Scp1 is palmitoylated intracellularly and localizes to the plasma membrane [10]. FG-1Adn-EGFP co-localized with Scp1-mCherry on the plasma membrane when FG-1Adn-EGFP was co-expressed in Scp1-mCherry-expressing cells. In contrast, when EGFP was co-expressed with Scp1-mCherry, EGFP was dispersed throughout the cell, and no co-localization with Scp1-mCherry on the plasma membrane was observed (Figure 5a). To perform a more detailed localization analysis of both molecules, the signal intensity was quantified. The results showed that FG-1Adn-EGFP co-localized with Scp1-mCherry on the plasma membrane, whereas EGFP did not (Figure 5b). These results strongly suggest that FG-1Adn interacts with Scp1 in cells.

To assess the physiological effects of FG-1Adn on Scp1, we analyzed the endogenous substrate response of Scp1 in cells transfected with FG-1Adn-EGFP. The phosphorylation level of CTDpS5 in RNAPII, a widely known endogenous substrate of Scp1, showed a marked increase in FG-1Adn-expressing H1299 cells compared to that in control cells in which EGFP was transfected (Figure 5c,d). Furthermore, the phosphorylation level of Ser473 of Akt (AktpS473), which is known to play a crucial role in nerve regeneration and is also an endogenous substrate of Scp1, showed a marked increase in FG-1Adn-expressing cells, as well as in CTDpS5, compared to that in control cells (Figure 5c,e). These results strongly suggest that FG-1Adn functions as an Scp1 inhibitor not only in vitro but also in cells.

## 3. Discussion

Scp1, which belongs to the FCP/SCP-type Ser/Thr protein phosphatase family, has been reported to play important roles in various biological processes, such as cell cycle regulation, signal transduction regulation, nerve gene silencing, and carcinogenesis [3,47]. Therefore, Scp1 inhibitors are expected to serve as anticancer agents and drugs for treating neurological diseases. The hydrophobic pocket of Scp1 is a promising target site for the development of Scp1-specific inhibitors [24,48]. Rabeprazole, a commercially available inhibitor targeting Scp1, has been reported to inhibit Scp isoforms by binding to these hydrophobic pockets close to the active center [24]. On the other hand, since rabeprazole was initially developed as a therapeutic agent for gastroesophageal reflux disease targeting the H^+^⁄K^+^ ATPase, the risk of side effects is a concern, as it is a molecule that acts on different targets. Therefore, the development of Scp1-specific inhibitors with high affinity and specificity is desirable for targeting the hydrophobic pocket of Scp1.

**Figure 6:**
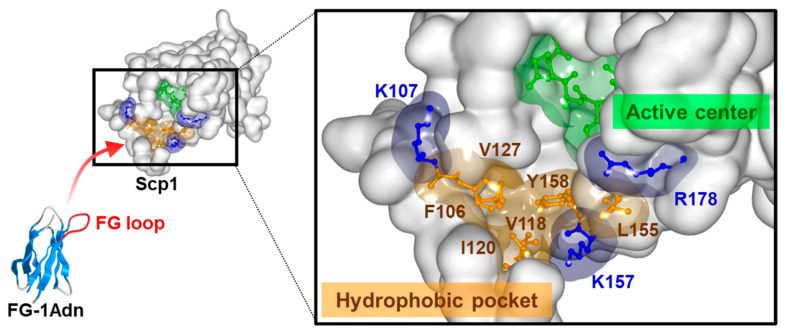
Binding model of FG-1Adn against Scp1. Hydrophobic pocket (orange) of Scp1 is located close to the DxDxT motif of the active center (green) in its catalytic domain as substrate recognition site. The hydrophobic pocket is surrounded by basic residues (blue), which are also expected to interact with the FG-1Adn.

In this study, we constructed a phage library containing the antibody-mimetic protein adnectin and identified Scp1-specific binding molecules using the library. In the adnectin-derived phage library, the FG loop of adnectin contained randomized amino acid residues, and this loop is known to contribute the most to sequence tolerance and binding to the target molecule [40,49]. Biopanning revealed that the FG loop peptide sequence from FG-1Adn, which was detected at the highest frequency when Scp1 bound to adnectin, showed the highest binding ability to Scp1. Ala scanning analysis of the heptad peptide (Phe_1_-Val_2_-Phe_3_-Gly_4_-Pro_5_-Asp_6_-Ala_7_) derived from the FG loop of FG-1Adn showed that Phe_1_-Val_2_-Phe_3_ and Pro_5_ play important roles in Scp1 binding (Figure 4a). CTDpS5-containing peptide (Tyr_1_-Ser_2_-Pro_3_-Thr_4_-pSer_5_-Pro_6_-Ser_7_) derived from RNAPII, known as an Scp1 substrate, has been reported to bind to the groove of the Scp1 active site through its Ser_2_-Pro_3_-Thr_4_-Ser_5_ residues in the sequence [23]. In particular, the hydrophobic interaction between Pro_3_ and the Scp1 hydrophobic pocket is important for the binding of CTDpS5 to Scp1. The fact that the Pro_3_ residues of FG-1Adn were highly conserved, even within the FG loop sequence of the Scp1-binding adnectin candidates identified in this study, also supports the notion that hydrophobic residues, including the Pro_3_ residues of FG-1, bind to the hydrophobic pocket close to the active center. In addition, the acidic residue Asp_6_ of FG-1Adn in the C-terminal region of the FG-1 peptide sequence may interact with basic residues located in the substrate-binding pocket of Scp1. Several basic residues were located around the substrate-binding hydrophobic pocket near the active center of Scp1 (Figure 6). These data suggest that electrostatic interactions through residues near these hydrophobic regions may also play important roles in the binding between FG-1Adn and Scp1. In addition, the Lineweaver–Burk plot analysis and the results of the competition study with rabeprazole supported that FG-1Adn binds to the hydrophobic region near the active center. In the Lineweaver–Burk plot analysis, the lines for the addition of different concentrations of FG-1Adn did not intersect on the *x*-axis but in the second quadrant. These results suggested that FG-1Adn functions as a non-competitive-like inhibitor and that FG-1Adn is an inhibitor with a higher affinity for the enzyme alone than for the substrate-enzyme complex.

Recently, it was reported that Scp1 dephosphorylates REST, c-Myc, and Akt as Scp1 substrates in cells [8,10,50]. The abnormal expression of REST is involved not only in carcinogenesis but also in neurological diseases, such as Parkinson’s disease and Huntington’s disease [13,14,51]. Therefore, specific inhibitors of Scp1 with high affinity and specificity may elucidate disease mechanisms and improve clinical therapy [52,53]. Rabeprazole is a commercially available Scp1 inhibitor; however, its affinity was relatively low, with IC_50_ values of 4 ± 0.7 μM and 9 ± 3 μM for *p*NPP and CTDpS5 as substrates, respectively [23]. We also reported on BeM12-1 as a peptidyl inhibitor for Scp1; however, it also failed to show strong inhibitory effects for Scp1, with an IC_50_ value of 100.4 μM for CTDpS5 as a substrate [26]. In contrast, FG-1Adn identified in this study exhibited high inhibitory activity against Scp1, with IC_50_ values of 112 ± 17 nM and 152 ± 26 nM for *p*NPP and CTDpS5, respectively. Thus, the inhibitory activity of FG-1Adn against Scp1 was 40–60 times higher than that of rabeprazole, even though they both bound to the hydrophobic pocket of the substrate binding site of Scp1 (Figure 4c,d). This may be attributed to the broad binding surface of FG-1Adn to Scp1 and the presence of multiple interaction sites between the enzyme and inhibitors compared with rabeprazole. It has also been reported that the cyclization of linear target-binding peptides or their presentation on stable scaffolds induces binding ability and selectivity toward the target [54,55]. Interestingly, FG-1Adn showed specific binding to Scp1; however, it showed weak binding to Scp2 and Scp3, and no binding to Fcp1 was observed among the FCP/SCP family members despite similar sequences and active center structures (Figure 3c). Thus, the high affinity and selectivity of FG-1Adn for Scp1 in this study suggest that the introduction of binding peptides for target proteins into adnectin molecules may be useful for improving their affinity and selectivity for the target molecules. In fact, the dissociation constant of FG-1Adn was comparable to Adnectin CT-322 (which binds to VEGFR-2) and Adnectin BMS-962476 (which binds to proprotein convertase subtilisin/kexin type 9), both of which are undergoing clinical trials [56,57,58]. This suggests that FG-1Adn possesses sufficient affinity to serve as an inhibitor with potential clinical applications. Thus, biopanning using a secondary library derived from FG-1Adn, in which a randomized sequence is introduced in the BC loop or the flanking region of the FG loop, could be applicable to identify further improved Scp1-specific inhibitors. Evaluation of the anti-tumor suppressive effects of FG-1Adn and its derivatives using endogenous Scp1-overexpressed cancer cells may demonstrate their usefulness as anti-tumor agents in the future.

One of the advantages of adnectin over antibodies is that it can function in reduced conditions, such as in cells, because adnectin contains no cysteine residue to form intramolecular disulfide bonds [43,44,45,59]. Scp1 is known to act as an intercellular protein phosphatase and has been reported to localize to the plasma membrane and Golgi apparatus through palmitoylation, a posttranslational lipid modification. In the present study, the expression of Scp1 in lung cancer-derived H1299 cells appeared not only in the cell but also in the plasma membrane, with a strong signal, indicating that Scp1 localizes to the plasma membrane, as previously reported [10]. Interestingly, FG-1Adn-EGFP was co-localized with Scp1 on the plasma membrane in H1299 cells, whereas EGFP alone was detected throughout the cell. These data strongly suggest that FG-1Adn interacts with Scp1 not only in vitro but also in cells. Furthermore, Western blotting analysis showed that the phosphorylation levels of CTDpS5 of RNAPII and AktpS473, both of which are endogenous substrates for Scp1, were enhanced in FG-1Adn-expressing cells compared to control cells, indicating that FG-1Adn has inhibitory activity against Scp1 in the cell. Further quantitative evaluation of phosphorylation levels of target proteins of Scp1 compared to the total protein in FG-1Adn treated cells will support and enhance the usefulness of FG-1Adn as an intracellular Scp1 inhibitor. In addition, it is expected that the introduction of cell-penetration peptide sequences, such as polyarginine, into FG-1Adn, will enable it to function as an Scp1 inhibitor that can spatiotemporally control cellular introduction [60,61]. 

Regarding the relationship between Scp1 and diseases, it has been reported that Scp1 contributes to the suppression of kidney and liver cancers via the dephosphorylation and stabilization of promyelocytic leukemia (PML) and c-myc [50,62,63]. On the other hand, it was recently reported that Scp1 stabilizes the zinc-finger type transcription factor SNAI1 (Snail), a known inducer of the epithelial–mesenchymal transition that promotes cancer metastasis and invasion in gastric cancer [22]. These findings suggest that Scp1-specific inhibitors may function as anticancer agents in gastric cancer. The detailed mechanisms underlying the opposing effects of Scp1 on carcinogenesis, that is, enhancement and suppression, have not yet been elucidated; however, FG-1Adn may be used as a molecular tool to clarify the function of Scp1 in carcinogenesis. Scp1 is also known to play an important role in gene silencing in neurons via the dephosphorylation and stabilization of REST. In addition, REST dysregulation has been implicated in several neurological diseases, including Huntington’s disease, Parkinson’s disease, and epilepsy [18,51]. Recently, it has also been reported that a decrease in the phosphorylation level of AktpS473, an Scp1 substrate, was observed in the area of nerve damage in mice, while an increase in its phosphorylation level was detected in the area of nerve regeneration [64]. These facts suggest that Scp1 inhibitors, including FG-1Adn, may be applied not only as anticancer agents but also as a novel approach for the treatment of neurological diseases and as an adjuvant molecule for nerve regeneration.

In summary, we constructed a phage library of antibody-mimetic adnectin-presenting molecules and identified FG-1Adn as an Scp1 inhibitor with high affinity and specificity. Binding and inhibition of Scp1 were observed not only in vitro but also in cells. Purified FG-1Adn is expected to be developed as a lead compound for the treatment of carcinogenesis and neurological diseases caused by the dysregulation of Scp1 through the development of a Drug Delivery System (DDS) in specific tissues and the establishment of a membrane permeation method.

## 4. Materials and Methods

### 4.1. Preparation of an Adnectin-Derived Phage Library

To construct a library containing randomized sequences of seven residues in the FG loops of adnectin, fragments containing randomized FG loops were created using adnectin cDNA as a template. The DNA fragments were amplified using primers FG7-fw: (5′-GCTGTCACTCTGTCGACA-NNKNNKNNKNNKNNKNNKNNK-TCTAGAAGCAAGCCAATTTC-3′) and FG7-rev: (5′-CTCCAAACTAGTTCTAGCGAATTCAAGC-TTATCG-3′). A DNA fragment encoding full-length adnectin randomizing seven residues in FG loops was prepared via PCR using a mix of DNA fragments as templates with primer FG7-rev. The PCR products were purified via agarose gel electrophoresis, digested with Sfi I and Spe I (TOYOBO, Osaka, Japan), and ligated to the linearized pKSTV-02 phagemid vector, which was kindly provided by Prof. Y. Ito (Kagoshima University, Kagoshima, Japan). The DNA was purified via phenol/chloroform treatment and ethanol precipitation and used for electro-transformation into *E. coli* TG-1 cells (Lucigen Co., Middleton, WI, USA). Titer analysis of the transformation revealed 0.83 × 10^7^ diversity. Transformed log-phase TG-1 cells were infected with M13KO7 helper phage (New England Biolabs, Ipswich, MA, USA) and cultured. To the culture supernatant collected by centrifugation, one-fifth of the volume of 20% polyethylene glycol 8000 solution containing 2.5 M NaCl was added. Phages were precipitated at 4 °C for 4 h and collected by centrifugation. The pellet was resuspended in PBS, and it was used following biopanning against Scp1. 

### 4.2. Screening of Adnectins against Scp1 Using FG loop Randomized Adnectin-Derived Phage Display Library

FG loop randomized adnectin-derived phage display library in binding buffer (20 mM Na-maleate (pH 5.5), 150 mM NaCl, 10 mM MgCl_2_, 0.5% BSA) was added to the BSA-coated ELISA plates to remove nonspecific binding phages to BSA. Wells of 96-well ELISA plates were coated with 100 µL of 2 µg/well recombinant Scp1 at 4 °C overnight. After the wells were blocked with BSA, the precleared phage library (2 × 10^10^ cfu/well) was added to the wells and incubated at R.T. for 1 h. The wells were then washed six times with 200 µL of wash buffer (20 mM Na-maleate (pH 5.5), 150 mM NaCl, 10 mM MgCl_2_). To isolate Scp1-specific phages, bound phages were eluted with 10 µg/well Scp1 in binding buffer to the well. 

### 4.3. Expression and Purification of Recombinant Adnectins

DNA fragment coding FG-1Adn phage was amplified via PCR using isolated clones as templates. The DNA fragment modified at the 3′-terminus by adding a nucleotide sequence encoding the 2 × Flag tag (2 × DYKDDDDK) was purified via agarose gel electrophoresis and ligated into pCold I vector (TaKaRa, Shiga, Japan) with Sal I and Xba I sites. The expression vector encoding the recombinant adnectins and Scp1 were transfected into the *E. coli* strain Rosetta (Novagen, Madison, WI, USA). The expressed proteins were purified with Talon affinity beads (TaKaRa, Shiga, Japan), as reported previously.

### 4.4. Binding Analysis of Adnectin-Derived Phages

Wells of 96-well plates were coated with 100 μL of 0.2 or 0.5 μg/well Scp1. After blocking with 0.5% BSA, isolated phages (1 × 10^10^ cfu/well) were added and incubated at R.T. for 1 h. After the washing steps with 200 μL of wash buffer (20 mM Na-maleate (pH 5.5), 150 mM NaCl, 10 mM MgCl_2_), anti-fd phage antibody (Sigma-Aldrich, St. Louis, MO, USA) followed by anti-rabbit HRP (Santa Cruz, Dallas, TX, USA) was employed. To detect bound phages, 100 μL of ABTS (2,2′-Azinobis [3-ethylbenzothiazoline-6-sulfonic acid]-diammonium salt)/H_2_O_2_ solution was added to each well, and the absorbance at 405 nm was measured using a ChroMate4300 microplate reader (Awareness Technology Chromate, Palm City, FL, USA).

### 4.5. Binding Analysis of Peptide-Conjugated Bacterial Alkaline Phosphatase

Bacterial alkaline phosphatase (BAP) chimeras with a peptide sequence identified as Scp1 specific FG loop of adnectin were generated by cloning synthetic oligonucleotides into pMY101, which was kindly provided by Dr. J. Rubin (National Institutes of Health, Bethesda, MD, USA). *E. coli* (DH5α) transformed with the peptide/BAP chimera constructs were grown in Luria broth (containing 100 mg/mL ampicillin). Bacterial broth normalized to BAP activity was added to the Scp1-coated wells, and the binding of chimeras to the Scp1 was measured with *p*-nitrophenyl phosphate (*p*NPP) (Sigma-Aldrich, St. Louis, MO, USA) as a substrate using a ChroMate4300 microplate reader. 

### 4.6. Binding Analysis of Recombinant Adnectins

A total of 0.5 μg/well of recombinant target proteins was coated on the wells of the 96-well plates. After blocking with 0.5% BSA, 0.05 μg/well of adnectins FG-1Adn and wtAdn, which was the original adnectin, were added to the well and incubated at R.T. for 1 h [35]. After 5 washes with 200 μL of wash buffer, anti-FLAG M2 antibody (Sigma-Aldrich, St. Louis, MO, USA) followed by anti-mouse-HRP (Cytiva, Marlborough, MA, USA) was employed. After the addition of ABTS/H_2_O_2_ as substrate, bound adnectin was measured at an absorbance of 405 nm using the ChroMate4300 microplate reader. For competition analysis, 100 µL of rabeprazole buffer (20 mM Na-maleate (pH 5.5), 150 mM NaCl, 10 mM MgCl_2_, 100 µM rabeprazole) was added to the well before adnectin binding.

### 4.7. pNPP Phosphatase Assay

In enzyme specificity analysis of FG-1Adn against FCP/SCP-type protein phosphatases, reaction buffer (20 mM Na-maleate (pH 5.5), 10 mM MgCl_2_) with FCP/SCP-type Ser/Thr protein phosphatase with GST-tag in 5′-terminus and Myc-His_6_ tag in 3′-terminus (Scp1; 0.083 mg/mL, Scp2; 0.027 mg/mL, Scp3; 0.104 mg/mL, Fcp1; 0.320 mg/mL) was incubated with 50 µL of 10 mM *p*NPP at 37 °C in 50 μL volume. In FG loop specificity analysis of recombinant adnectin against Scp1, 10 nM of His_6_-Scp1 was incubated with reaction buffer. When adnectin was included, phosphatase and adnectin were preincubated in a reaction buffer on ice for 30 min. For PMD-24 adnectin, which was reported to be an adnectin-targeting oncogenic, PPM1D was used as CtrlAdn [43]. After the reactions (Scps; 7 min, Fcp1; 60 min), the reactions were quenched by adding 70 μL of 2% SDS, and the absorbance at 410 nm was measured. The IC_50_ value was calculated using log(inhibitor) versus response variable slope equation with GraphPad Prism8 software (Equation (1))

Y = 100/{1 + 10^(log IC_50_-X)*Hillslope)^} (1)

where X is the adnectin concentration, and Y represents the corresponding percentage values of enzyme activity. For enzyme kinetic analysis, the mode of inhibition was analyzed using a Lineweaver–Burk plot (Equation (2)).

1/v = (*K*_M_/*V*_max_)*(1/[S]) + (1/*V*_max_) (2)

where [S] is the *p*NPP concentration. *K*_M_ and *V*_max_ were calculated using the Michaelis–Menten equation with GraphPad Prism8 software.

### 4.8. Malachite Green Assay

Reaction buffer (20 mM Na-maleate (pH 5.5), 10 mM MgCl_2_) with 20 µM CTDpS5 peptide (Ac-SPSYSPTpSPS-NH_2_) was incubated with 30 µL of 10 nM His_6_-Scp1 at 37 °C in 50 μL volume. When adnectin was included, Scp1 and adnectin were preincubated in Scp1 buffer on ice for 30 min. After 7 min of reaction, the reactions were quenched by adding 100 μL of BIOMOL GREEN reagent (Enzo Life Science, Plymouth, PA, USA) and incubated at R.T. for 30 min. The release of free inorganic phosphate was determined by measuring the absorbance at 620 nm. The IC_50_ value was calculated using the log(inhibitor) versus response variable slope equation with GraphPad Prism8 software (Equation (1)).

### 4.9. Biolayer Interferometry BLItz System Assay

To analyze the binding affinity between FG-1Adn and Scp1, SARSTORIUS streptavidin biosensors were prehydrated for 10 min. The biotinylated FG-1Adn by Biotin Labeling Kit-SH (Biotin Labeling Kit-SH, Chemical Dojin Co., Ltd., Tokyo, Japan) were loaded onto the biosensors, which were equilibrated in 20 mM Na-maleate (pH 5.5), 150 mM NaCl, 10 mM MgCl_2_, and 0.05% Tween for 120 s and then exposed to solutions containing 250 nM Scp1 for 120 s as the association step. The biosensors were then transferred to a maleate buffer for a 120 s dissociation step. Data were analyzed using the data analysis software BLItz Pro 1.3.0.5 Software (Fortebio, Inc., Menlo Park, CA, USA), and the *K*_D_ value between FG-1Adn and Scp1 was also calculated using a standard 1:1 Langmuir binding model.

### 4.10. Subcellular Localization Analysis of Transfected Adnectin

H1299 cells were cultured in Dulbecco’s modified Eagle’s medium (DMEM) supplemented with 10% heat-inactivated fetal bovine serum (FBS) at 37 °C in 5% CO_2_. 1.5 × 10^5^ cells of H1299 cells were plated in a 35 mm dish with 2 mL of medium and incubated for 24 h. The transfection of H1299 cells in a 35 mm dish with 2 μg of each expression construct using X-tremeGENE HP DNA Transfection Reagent (Roche, Basel, Switzerland) was performed according to the manufacturer’s instructions. Then, 48 h after the transfection, the cell culture medium was exchanged to phenol red-free DMEM medium, and the subcellular localization of Scp1-mCherry and FG-1Adn-EGFP was analyzed using a BZ-X800 fluorescence microscope (Keyence, Osaka, Japan).

### 4.11. Western Blotting

For transfected FG-1 samples, 1.5 × 10^5^ cells of H1299 cells were plated in a 35 mm dish with 2 mL of medium and incubated for 24 h. The cells were harvested 48 h after transfection, and the cell lysate was prepared from cultured cells using (1 × phosphatase inhibitor (Nacalai Tesque, Kyoto, Japan), 50 mM Tris-HCl (pH 7.5) and 500 mM NaCl, 1% TritonX-100) with a 1% protease inhibitor cocktail (Nacalai Tesque, Kyoto, Japan). Normalized protein extracts were used for analysis via SDS–PAGE and immunoblotting with Immobilon-P membranes (Millipore, Burlington, MA, USA). Anti-CTDpS5 antibody (Abcam, Cambridge, UK), anti-p-Akt1 antibody (Santa Cruz, Dallas, TX, USA), and anti-GAPDH antibody (Santa Cruz, Dallas, TX, USA) were used as primary antibodies and incubated with the transferred membranes at 4 °C overnight. After washing the membranes, the solutions of anti-mouse-HRP (Cytiva, Marlborough, MA, USA) were added to the membranes and incubated at room temperature for 30 min. The membranes were visualized with ECL reagent (GE healthcare, Chicago, IL, USA) using C-Digit blot scanner (MS Techno Systems Inc., Tokyo, Japan).

### 4.12. Statistical Analysis

All of the statistical analysis were conducted with GraphPad Prism version 8 software. Data are presented as means ± S.D. and SEM. Data shown in the study were obtained at least from three times independent experiments with some exceptions: (binding analysis of BAP (Figure 2b), inhibitory analysis (Figure 4a), competition analysis (Figure 4e), and enzyme inhibitory kinetic analysis (Figure 4d). Statistical significance was determined using an unpaired, two-tailed Student’s t-test or two-way ANOVA as appropriate.

## Figures and Tables

**Table 1:** FG loop sequences obtained via biopanning using the FG randomized adnectin-derived phage display library against Scp1 in 3rd and 4th round. The sequences of isolated clones and their frequencies were shown.

Name	Sequence	Frequency	
		3rd Round	4th Round
FG-1	FVFGPDA	1	12
FG-2	LLEPEDW	1	4
FG-3	RPESWGI		1
FG-4	TFQCSIL		1
FG-5	LFEPADR	1	
Total		3	18

## Data Availability

The data that support the findings of this study are available from the corresponding author, [Y.C.], upon reasonable request.

## References

[1] Olsen J.V., Blagoev B., Gnad F., Macek B., Kumar C., Mortensen P., Mann M. (2006). Global, in vivo, and site-specific phosphorylation dynamics in signaling networks. Cell.

[2] Shi Y. (2009). Serine/threonine phosphatases: Mechanism through structure. Cell.

[3] Zhang Y., Kim Y., Genoud N., Gao J., Kelly J.W., Pfaff S.L., Gill G.N., Dixon J.E., Noel J.P. (2006). Determinants for dephosphorylation of the RNA polymerase II C-terminal domain by Scp1. Mol. Cell.

[4] Yeo M., Lin P.S., Dahmus M.E., Gill G.N. (2003). A novel RNA polymerase II C-terminal domain phosphatase that preferentially dephosphorylates serine 5. J. Biol. Chem..

[5] Harikrishna R., Kim H., Noh K., Kim Y.J. (2014). The diverse roles of RNA polymerase II C-terminal domain phosphatase SCP1. BMB Rep..

[6] Zhu Y., Lu Y., Zhang Q., Liu J.J., Li T.J., Yang J.R., Zeng C., Zhuang S.M. (2012). MicroRNA-26a/b and their host genes cooperate to inhibit the G1/S transition by activating the pRb protein. Nucleic Acids Res..

[7] Wrighton K.H., Willis D., Long J., Liu F., Lin X., Feng X.H. (2006). Small C-terminal domain phosphatases dephosphorylate the regulatory linker regions of Smad2 and Smad3 to enhance transforming growth factor-beta signaling. J. Biol. Chem..

[8] Burkholder N.T., Mayfield J.E., Yu X., Irani S., Arce D.K., Jiang F., Matthews W.L., Xue Y., Zhang Y.J. (2018). Phosphatase activity of small C-terminal domain phosphatase 1 (SCP1) controls the stability of the key neuronal regulator RE1-silencing transcription factor (REST). J. Biol. Chem..

[9] Gervasi N.M., Dimtchev A., Clark D.M., Dingle M., Pisarchik A.V., Nesti L.J. (2021). C-terminal domain small phosphatase 1 (CTDSP1) regulates growth factor expression and axonal regeneration in peripheral nerve tissue. Sci. Rep..

[10] Liao P., Wang W., Li Y., Wang R., Jin J., Pang W., Chen Y., Shen M., Wang X., Jiang D. (2017). Palmitoylated SCP1 is targeted to the plasma membrane and negatively regulates angiogenesis. Elife.

[11] Yeo M., Lee S.K., Lee B., Ruiz E.C., Pfaff S.L., Gill G.N. (2005). Small CTD phosphatases function in silencing neuronal gene expression. Science.

[12] Ashton N.J., Hye A., Leckey C.A., Jones A.R., Gardner A., Elliott C., Wetherell J.L., Lenze E.J., Killick R., Marchant N.L. (2017). Plasma REST: A novel candidate biomarker of Alzheimer’s disease is modified by psychological intervention in an at risk population. Transl. Psychiatry.

[13] Kawamura M., Sato S., Matsumoto G., Fukuda T., Shiba-Fukushima K., Noda S., Takanashi M., Mori N., Hattori N. (2019). Loss of nuclear REST/NRSF in aged-dopaminergic neurons in Parkinson’s disease patients. Neurosci. Lett..

[14] de Souza J.M., Abd-Elrahman K.S., Ribeiro F.M., Ferguson S.S.G. (2020). mGluR5 regulates REST/NRSF signaling through N-cadherin/β-catenin complex in Huntington’s disease. Mol. Brain.

[15] Spencer E.M., Chandler K.E., Haddley K., Howard M.R., Hughes D., Belyaev N.D., Coulson J.M., Stewart J.P., Buckley N.J., Kipar A. (2006). Regulation and role of REST and REST4 variants in modulation of gene expression in in vivo and in vitro in epilepsy models. Neurobiol. Dis..

[16] Wu Y., Evers B.M., Zhou B.P. (2009). Small C-terminal domain phosphatase enhances snail activity through dephosphorylation. J. Biol. Chem..

[17] Qian W., Li Q., Wu X., Li W., Li Q., Zhang J., Li M., Zhang D., Zhao H., Zou X. (2020). Deubiquitinase USP29 promotes gastric cancer cell migration by cooperating with phosphatase SCP1 to stabilize Snail protein. Oncogene.

[18] Garcia-Manteiga J.M., D’Alessandro R., Meldolesi J. (2019). News about the Role of the Transcription Factor REST in Neurons: From Physiology to Pathology. Int. J. Mol. Sci..

[19] Glaviano A., Foo A.S.C., Lam H.Y., Yap K.C.H., Jacot W., Jones R.H., Eng H., Nair M.G., Makvandi P., Geoerger B. (2023). PI3K/AKT/mTOR signaling transduction pathway and targeted therapies in cancer. Mol. Cancer.

[20] Liu T., Wang W., Li X., Chen Y., Mu F., Wen A., Liu M., Ding Y. (2023). Advances of phytotherapy in ischemic stroke targeting PI3K/Akt signaling. Phytother. Res..

[21] Sharma A., Mehan S. (2021). Targeting PI3K-AKT/mTOR signaling in the prevention of autism. Neurochem. Int..

[22] Kanlaya R., Kapincharanon C., Fong-Ngern K., Thongboonkerd V. (2022). Induction of mesenchymal-epithelial transition (MET) by epigallocatechin-3-gallate to reverse epithelial-mesenchymal transition (EMT) in SNAI1-overexpressed renal cells: A potential anti-fibrotic strategy. J. Nutr. Biochem..

[23] Zhang M., Cho E.J., Burstein G., Siegel D., Zhang Y. (2011). Selective inactivation of a human neuronal silencing phosphatase by a small molecule inhibitor. ACS Chem. Biol..

[24] Medellin B., Yang W., Konduri S., Dong J., Irani S., Wu H., Matthews W.L., Zhang Z.Y., Siegel D., Zhang Y. (2022). Targeted Covalent Inhibition of Small CTD Phosphatase 1 to Promote the Degradation of the REST Transcription Factor in Human Cells. J. Med. Chem..

[25] Otsubo K., Yoneda T., Kaneko A., Yagi S., Furukawa K., Chuman Y. (2018). Development of a Substrate Identification Method for Human Scp1 Phosphatase Using Phosphorylation Mimic Phage Display. Protein Pept. Lett..

[26] Yoshida T., Yamazaki K., Imai S., Banno A., Kaneko A., Furukawa K., Chuman Y. (2019). Identification of a Specific Inhibitor of Human Scp1 Phosphatase Using the Phosphorylation Mimic Phage Display Method. Catalysts.

[27] Mizunuma M., Kaneko A., Imai S., Furukawa K., Chuman Y. (2020). Methods for Identification of Substrates/Inhibitors of FCP/SCP Type Protein Ser/Thr Phosphatases. Processes.

[28] Williams M.P., Pounder R.E. (1999). Review article: The pharmacology of rabeprazole. Aliment. Pharmacol. Ther..

[29] Saeed A.F., Wang R., Ling S., Wang S. (2017). Antibody Engineering for Pursuing a Healthier Future. Front. Microbiol..

[30] Simeon R., Chen Z. (2018). In vitro-engineered non-antibody protein therapeutics. Protein Cell..

[31] Sha F., Salzman G., Gupta A., Koide S. (2017). Monobodies and other synthetic binding proteins for expanding protein science. Protein Sci..

[32] Liu M., Li L., Jin D., Liu Y. (2021). Nanobody-A versatile tool for cancer diagnosis and therapeutics. Wiley Interdiscip. Rev. Nanomed. Nanobiotechnol..

[33] Plückthun A. (2015). Designed ankyrin repeat proteins (DARPins): Binding proteins for research, diagnostics, and therapy. Annu. Rev. Pharmacol. Toxicol..

[34] Rothe C., Skerra A. (2018). Anticalin^®^ Proteins as Therapeutic Agents in Human Diseases. BioDrugs.

[35] Koide A., Bailey C.W., Huang X., Koide S. (1998). The fibronectin type III domain as a scaffold for novel binding proteins. J. Mol. Biol..

[36] Park S.H., Park S., Kim D.Y., Pyo A., Kimura R.H., Sathirachinda A., Choy H.E., Min J.J., Gambhir S.S., Hong Y. (2015). Isolation and Characterization of a Monobody with a Fibronectin Domain III Scaffold That Specifically Binds EphA2. PLoS ONE.

[37] Weidle U.H., Auer J., Brinkmann U., Georges G., Tiefenthaler G. (2013). The emerging role of new protein scaffold-based agents for treatment of cancer. Cancer Genom. Proteom..

[38] Koide A., Wojcik J., Gilbreth R.N., Hoey R.J., Koide S. (2012). Teaching an old scaffold new tricks: Monobodies constructed using alternative surfaces of the FN3 scaffold. J. Mol. Biol..

[39] Batori V., Koide A., Koide S. (2002). Exploring the potential of the monobody scaffold: Effects of loop elongation on the stability of a fibronectin type III domain. Protein Eng..

[40] Cheung L.S., Shea D.J., Nicholes N., Date A., Ostermeier M., Konstantopoulos K. (2015). Characterization of monobody scaffold interactions with ligand via force spectroscopy and steered molecular dynamics. Sci. Rep..

[41] Richards J., Miller M., Abend J., Koide A., Koide S., Dewhurst S. (2003). Engineered fibronectin type III domain with a RGDWXE sequence binds with enhanced affinity and specificity to human alphavbeta3 integrin. J. Mol. Biol..

[42] Karatan E., Merguerian M., Han Z., Scholle M.D., Koide S., Kay B.K. (2004). Molecular recognition properties of FN3 monobodies that bind the Src SH3 domain. Chem. Biol..

[43] Ikeura M., Tashiro H., Yamagata Y., Saito H., Kobayashi T., Mizunuma M., Yamazaki K., Baba K., Furukawa K., Chuman Y. (2022). Development of Antibody-like Proteins Targeting the Oncogenic Ser/Thr Protein Phosphatase PPM1D. Processes.

[44] Guntas G., Lewis S.M., Mulvaney K.M., Cloer E.W., Tripathy A., Lane T.R., Major M.B., Kuhlman B. (2016). Engineering a genetically encoded competitive inhibitor of the KEAP1-NRF2 interaction via structure-based design and phage display. Protein Eng. Des. Sel..

[45] Wallon L., Khan I., Teng K.W., Koide A., Zuberi M., Li J., Ketavarapu G., Traaseth N.J., O’Bryan J.P., Koide S. (2022). Inhibition of RAS-driven signaling and tumorigenesis with a pan-RAS monobody targeting the Switch I/II pocket. Proc. Natl. Acad. Sci. USA.

[46] Kondo T., Iwatani Y., Matsuoka K., Fujino T., Umemoto S., Yokomaku Y., Ishizaki K., Kito S., Sezaki T., Hayashi G. (2020). Antibody-like proteins that capture and neutralize SARS-CoV-2. Sci. Adv..

[47] Rallabandi H.R., Ganesan P., Kim Y.J. (2020). Targeting the C-Terminal Domain Small Phosphatase 1. Life.

[48] Kamenski T., Heilmeier S., Meinhart A., Cramer P. (2004). Structure and mechanism of RNA polymerase II CTD phosphatases. Mol. Cell.

[49] Wojcik J., Lamontanara A.J., Grabe G., Koide A., Akin L., Gerig B., Hantschel O., Koide S. (2016). Allosteric Inhibition of Bcr-Abl Kinase by High Affinity Monobody Inhibitors Directed to the Src Homology 2 (SH2)-Kinase Interface. J. Biol. Chem..

[50] Wang W., Liao P., Shen M., Chen T., Chen Y., Li Y., Lin X., Ge X., Wang P. (2016). SCP1 regulates c-Myc stability and functions through dephosphorylating c-Myc Ser62. Oncogene.

[51] Song Z., Zhao D., Zhao H., Yang L. (2015). NRSF: An angel or a devil in neurogenesis and neurological diseases. J. Mol. Neurosci..

[52] Palm K., Metsis M., Timmusk T. (1999). Neuron-specific splicing of zinc finger transcription factor REST/NRSF/XBR is frequent in neuroblastomas and conserved in human, mouse and rat. Brain Res. Mol. Brain Res..

[53] Zuccato C., Tartari M., Crotti A., Goffredo D., Valenza M., Conti L., Cataudella T., Leavitt B.R., Hayden M.R., Timmusk T. (2003). Huntingtin interacts with REST/NRSF to modulate the transcription of NRSE-controlled neuronal genes. Nat. Genet..

[54] Ngambenjawong C., Pineda J.M., Pun S.H. (2016). Engineering an Affinity-Enhanced Peptide through Optimization of Cyclization Chemistry. Bioconjug. Chem..

[55] Mihara E., Watanabe S., Bashiruddin N.K., Nakamura N., Matoba K., Sano Y., Maini R., Yin Y., Sakai K., Arimori T. (2021). Lasso-grafting of macrocyclic peptide pharmacophores yields multi-functional proteins. Nat. Commun..

[56] Mamluk R., Carvajal I.M., Morse B.A., Wong H., Abramowitz J., Aslanian S., Lim A.C., Gokemeijer J., Storek M.J., Lee J. (2010). Anti-tumor effect of CT-322 as an adnectin inhibitor of vascular endothelial growth factor receptor-2. MAbs.

[57] Schiff D., Kesari S., de Groot J., Mikkelsen T., Drappatz J., Coyle T., Fichtel L., Silver B., Walters I., Reardon D. (2015). Phase 2 study of CT-322, a targeted biologic inhibitor of VEGFR-2 based on a domain of human fibronectin, in recurrent glioblastoma. Investig. New Drugs.

[58] Mitchell T., Chao G., Sitkoff D., Lo F., Monshizadegan H., Meyers D., Low S., Russo K., DiBella R., Denhez F. (2014). Pharmacologic profile of the Adnectin BMS-962476, a small protein biologic alternative to PCSK9 antibodies for low-density lipoprotein lowering. J. Pharmacol. Exp. Ther..

[59] Yu X., Yang Y.P., Dikici E., Deo S.K., Daunert S. (2017). Beyond Antibodies as Binding Partners: The Role of Antibody Mimetics in Bioanalysis. Annu. Rev. Anal. Chem..

[60] Futaki S., Nakase I. (2017). Cell-Surface Interactions on Arginine-Rich Cell-Penetrating Peptides Allow for Multiplex Modes of Internalization. Acc. Chem. Res..

[61] Habault J., Poyet J.L. (2019). Recent Advances in Cell Penetrating Peptide-Based Anticancer Therapies. Molecules.

[62] Lin Y.C., Lu L.T., Chen H.Y., Duan X., Lin X., Feng X.H., Tang M.J., Chen R.H. (2014). SCP phosphatases suppress renal cell carcinoma by stabilizing PML and inhibiting mTOR/HIF signaling. Cancer Res..

[63] Krasnov G.S., Puzanov G.A., Dashinimaev E.B., Vishnyakova K.S., Kondratieva T.T., Chegodaev Y.S., Postnov A.Y., Senchenko V.N., Yegorov Y.E. (2023). Tumor Suppressor Properties of Small C-Terminal Domain Phosphatases in Clear Cell Renal Cell Carcinoma. Int. J. Mol. Sci..

[64] Zhang K.L., Li S.M., Hou J.Y., Hong Y.H., Chen X.X., Zhou C.Q., Wu H., Zheng G.H., Zeng C.T., Wu H.D. (2023). Elabela, a Novel Peptide, Exerts Neuroprotective Effects Against Ischemic Stroke Through the APJ/miR-124-3p/CTDSP1/AKT Pathway. Cell. Mol. Neurobiol..

